# Biogenic synthesis of zinc oxide-impregnated *Musa acuminata* peel composite as an efficient lignocellulosic macromolecular sieve for enhanced eradication of Congo red dye from wastewater

**DOI:** 10.1039/d6ra01452f

**Published:** 2026-05-27

**Authors:** Muhammad Raza, Rabia Rehman, Ghufrana Samin, Amara Dar, Zahrah T. Al-thagafi, Eman A. Al-Abbad, Mehwish Akram, Fadi Alakhras

**Affiliations:** a School of Chemistry, University of the Punjab, Quaid-e-Azam Campus Lahore-54590 Pakistan chemistofpu@gmail.com g.samin@uet.edu.pk amara.chem@pu.edu.pk rabia.chem@pu.edu.pk; b Department of Basic Sciences and Humanities, University of Engineering and Technology, Faisalabad Campus Pakistan; c Department of Chemistry, College of Science, Taif University P.O. Box 11099 Taif 21944 Saudi Arabia; d Department of Chemistry, College of Science, Imam Abdulrahman Bin Faisal University P.O. Box 1982 Dammam 31441 Saudi Arabia ealabbad@iau.edu.sa; e Institute of Geology, University of the Punjab, Quaid-e-Azam Campus Lahore Pakistan mehwish.geo@pu.edu.pk; f Chemistry Department, College of Science, Jerash University Jerash Jordan fadialakhras@gmail.com

## Abstract

Uncontrolled urbanization and industrialization, coupled with population growth, have increased water pollution in the world. Banana peel powder was used as biosorbent in two forms, through artificial dyeing in the industry and other coloring agents in industrial discharge. This study examines the efficiency of zinc oxide-mediated agro-waste material made of *Musa acuminata* (banana) peels for the adsorption of Congo red (CR) dye in industrial effluents. Banana peel powder was use as biosorbent in two forms, namely, in their natural state (UMP) and ZnO-treated form (TMP). The optimal conditions for the removal of dye in the batch process were determined for UMP at pH 6 and TMP at pH 5.8. The optimal dosage for the adsorbents was 1.2 g UMP and 1.2 g TMP. The Temkin model provided the most acceptable alignment to the experimental equilibrium results among the other isothermal models, with *R*^2^ values of 0.9922 for UMP, and Langmuir was the best model with an *R*^2^ value of 0.9482 for TMP. Although the isothermal studies also showed good correlation coefficients, the Temkin model provided the best fit. The biosorption behavior was best described by pseudo second order kinetics, with *R*^2^ = 0.999 for UMP and *R*^2^ = 0.9994 for TMP. The values of the thermodynamic parameters Δ*G*°, Δ*S*° and Δ*H*° are −17.175 kJ mol^−1^ at 303 K, −3.798 kJ mol^−1^ and -20.628 kJ mol^−1^ for TMP and −16.964 kJ mol^−1^ at 303 K, −20.026 J mol^−1^ K^−1^, and −25.943 kJ mol^−1^ for UMP, respectively, indicating their exothermic and spontaneous nature.

## Abstract 2

Upcycling of banana peels agro-waste to control dye pollution by green chemistry.

## Graphical abstract

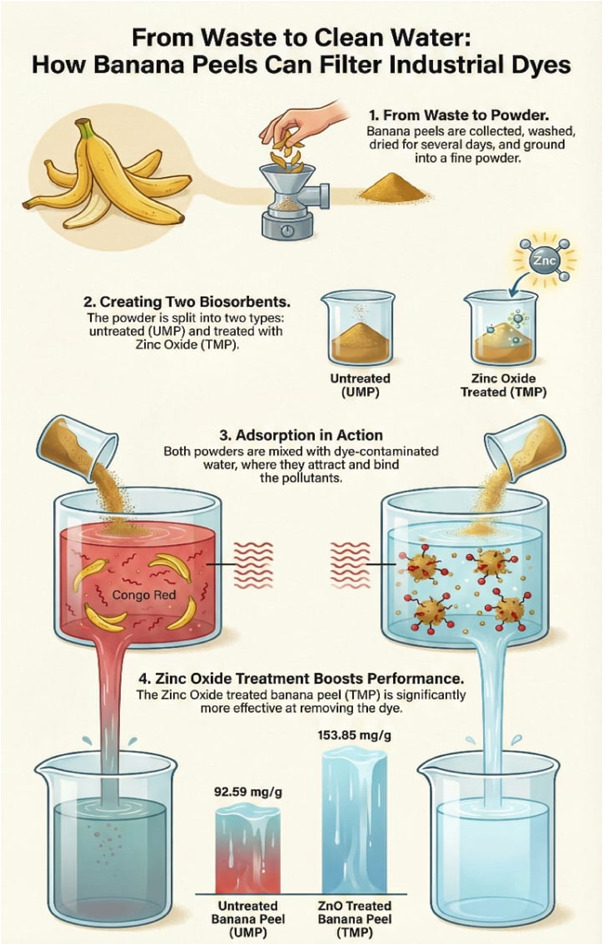

## 1 Introduction

Industrialization and urbanization have increased water usage, along with population growth. The most notable effect is the production of chromatic pollutants by the textiles, papers and leather industries, which have an adverse effect on flora, fauna and aquatic life.^1^ Dyes can be classified into cationic, non-ionic and anionic (acidic, reactive, and direct dyes). Reactive azo dye species, including methyl red, methyl orange, Congo red, direct red, and phenyl orange, are estimated to account for over 50 percent of the total consumption in industries over the past several decades.^2^ It is a fact that dyes find wide application in the industry; owing to their aromatic nature and light/heat/oxidant resistant quality, dyes are hard to scrape off sludge water. Natural compounds or human-made compounds can be used as colorants. The manufactured colorants are water-soluble, aromatic, and highly dispersible in nature. A few examples of industries using dyes made by humans include paper, painting, textiles and leather. The efficiency of color stabilization in the dyeing process is relatively low, with 10–15% of the unfixed dye subsequently drained to water during textile manufacturing operations.^3^ Waste water released by different industries and households contains large amounts of dangerous inorganic (heavy metal and metalloid ions) and organic (synthetic dyes, PAHs, *etc.*) compounds, threatening the environment and all the living creatures.^4^ The use of synthetic dyes is essential because they can dye different materials and clothes, giving them a beautiful appearance. These dyes play an important role in many industrial processes. The largest and most recognized user of dyes among these industries is the textile industry, with about 700 000 tons of annual usage.^5^

Several studies have reported that the textile industry consumes many dyes annually, including Rh–B, VB, RB, IR, Car Red 120, and Congo red. Approximately 20% (about one-third) of these dyes are lost during synthesis and processing and are released into the wastewater.^6^ Such effluents with dye-pollutants contain colorful pigments that are highly toxic, non-biodegradable and harmful to life forms. It is estimated that about 10 000 various types of dyes are manufactured, totaling more than 700 000 tons per annum as per figures of the color index. The popularity and widespread use of these synthetic dyes can be explained by their outstanding coloring strength, low cost, ease of acquiring the raw material, simple preparation, good fastness and ability to span the entire spectrum of shades. The most common commercial dyes are azo dyes. Other dye classes include phthalocyanine, anthraquinone, polymethines and aryl-carbonium.^7^

Various treatment methods have been investigated to eliminate dyes from effluent water. These methods include membrane separation, coagulation–flocculation, chemical oxidation, adsorption, chemical reduction, photo-degradation, electrochemical oxidation, Fenton oxidation, and biological treatment.^8^ Each of these techniques has its advantages and disadvantages. The adsorption operation has attracted interest because it is simple, cheap and allows the recycling of adsorbent materials.^9^ The enhanced removal of contaminants in wastewater, such as dyes and pigments, has attracted attention in the field of water pollution. One important method for eliminating pollutants is adsorption, which utilizes solid materials commonly referred to as adsorbents/biosorbents, based on the source of supply.^10^

Various physiochemical processes for addressing this issue include absorption, chemical precipitation, chemical coagulation, electrochemical reduction, filtration, and oxidation. Nevertheless, these approaches have disadvantages because they are costly and inefficient in removing dyes.^11^ These techniques generate large amounts of secondary sludge, leading to wastewater pollution. One of the most basic and easiest methods of treating water is by adsorbing dyes on the surface of nanocomposites or nanomaterials in order to eliminate dyes in the water media owing to easy-to-operate instrumentation, economical cost and high removal capacity.^12^ Nevertheless, since there are no biological entities in the composition of the adsorbent, the latter lacks a high degree of biocompatibility. Due to these constraints, researchers have turned their attention to biological wastewater remediation. In addition to dye removal in aqueous media, the removal of dyes and metals has also been reported in several studies to enhance drinking water quality.^13^ As suggested in previous reports, there are numerous adsorption techniques, but biological adsorption treatment is quite intriguing for the realization of safe treatment methods. As is evident, biological adsorption treatment is a recent strategy in which scientists employ the use of algae, plants, yeasts and agro-waste to eliminate dyes or transform hazardous compounds into mild substances.^14^ The benefits of this technique include biodegradability, high adsorbent content, rapidity, immediate resource availability, and cost-effective consumption. Several parameters, like biomass quantity, contact time, initial color concentration, pH, rotation speed, and temperature, have also been considered for dye removal efficiency using algae. The effect of temperature on CR elimination by *Chlorella candida* algae was investigated. This study concluded that 50 ppm of CR dye in a hydrous solution was effectively removed at 35 °C for 216 h using the algae.

In addition, we examined various isothermal models, after which the Freundlich model best fitted the data derived from the dye removal. It was reported to eliminate dyes like crystal violet, methylene blue and safranin out of the sea water by means of raw biomass and moisture-free biomass of microalgae *P*. *tricornutum*. The outcomes of dye elimination factors (contact time, pH and dye concentration) provide maximum dye elimination capacity, which was associated with crystal violet, and no characteristic difference between raw and moisture-free microalgae biomass was observed.^15^

Many studies have evaluated the efficiency of dye removal using various biochars made from agricultural wastes in the aqueous phase. Biochar is a perfect adsorbent in which biomass can be converted through a low pyrolysis procedure for the efficient, sustainable and environmentally friendly adsorption of dyes. Conversely, low-cost adsorbents can be utilized, like banana peels. Conversely, low-cost adsorbents can be utilized in banana peels. In fruit peels, lignin, cellulose, hemicelluloses, carboxyl, hydroxyl, and pectin materials are usually present, and these improve the interactions between the dye and the absorbent. Every year, the world produces about 250 million tons of bananas. After a banana is consumed, its peel has no further use and is freely available. Banana peel is thrown as market waste, and it poses environmental issues. Consequently, agricultural residues containing carbon end up in landfills and can potentially result in greenhouse emissions. Therefore, it would be financially viable to produce biochar from wastes, especially agricultural wastes, minimizing waste, and creating wealth.^16^

Different types of metal oxide-coated biochar nanocomposites, such as cellulosic material-coated zinc oxide-biochar, including zinc oxide-coated nanocomposites and zinc oxide-treated nanocomposites, were prepared from the biomass of different materials. The benefits of larger surface areas and an increased number of reactive sites, enhanced dispersion, a strong electronic interaction between ZnO and biomaterials, and further extended photocatalytic activity are the benefits of ZnO-coated bio-nanocomposites. Porous carbon-supported ZnO nanocomposites have been produced in recent years, widening the number of raw materials and improving photo catalysis. Although the real potential of ZnO bio-nanocomposite has not been fully exploited, its microstructure has demonstrated the potential to enhance photocatalytic degradation by supplementing biocomposite. Therefore, the compositions of ZnO nanoparticles and biocomposite have significant potential for the photosensitized degradation of organic pollutants.^17^

CR was selected as the dye to be extracted from an aqueous solution using banana peel (*Musa acuminata*) powder in this study. The adsorbent was characterized using FT-IR, EDX and SEM. To evaluate the impact of experimental conditions, factors like pH, dose of adsorbent, time of contact, concentration of dye at initial level and temperature were investigated. Additionally, the mechanism of adsorption CR should be investigated using the kinetics models provided by the pseudo 1st order, pseudo 2nd order, and thermodynamic studies. The regeneration of biowaste was carried out as the final step in this work.

## 2 Material and methods

### 2.1 Materials

The glass equipment utilized in the experiment was washed with D.W and H_2_CrO_4_ and then heated using an electric furnace for over 60 min at 60 degrees Celsius. The glass apparatus is made with Pyrex. Table 1 lists the chemicals and instruments used.

**Table 1:** Instrumentations and chemicals

Category	Materials/instruments
Chemicals	Hydrochloric acid
	Hydrogen peroxide
	Potassium iodide
	Sodium hydroxide
	Potassium chloride
	Starch
	Sulfuric acid
	Zinc oxide
	Methylene blue
Instruments	Scanning electron microscope
	Infrared electron microscope
	Spectrophotometer

### 2.2 Methodology

#### 2.2.1 Biosorbent

**Fig. 1:**
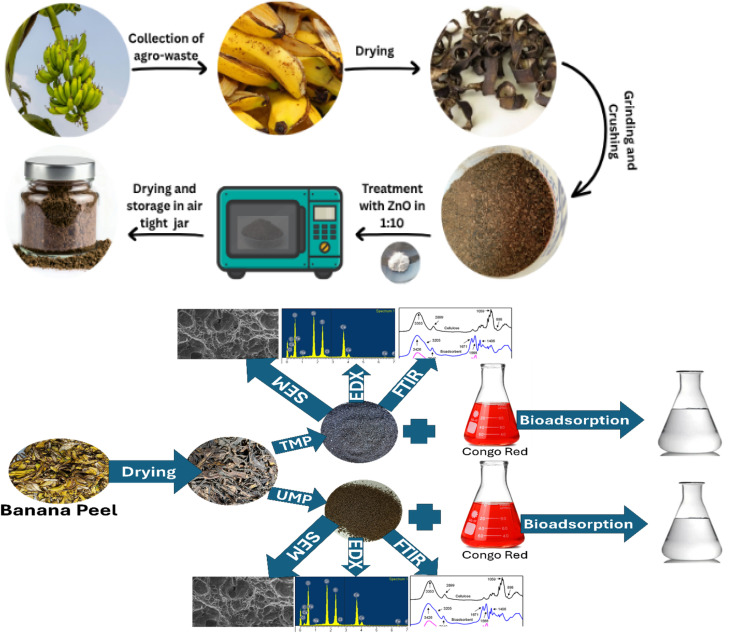
Representation diagram showing the preparation of the biosorbent and the scheme of work.

Banana peels were collected from the market and scrubbed with water, followed by D. W to eradicate the last bits of dirt. The peels were cleaned carefully and then cut into small pieces before being exposed to the sun for at least six to seven days until they dried. The dried peel was converted to powder in a spin mill with double blades when completely dry. It was further ground into an incredibly small size using a grinder and sieved through a 60-mesh sieve. The powder sorbent was dipped in water and then placed in a 1000 mL beaker with 15–20 mL of H_2_O_2_ for decolorization. Then, the adsorbent was permitted to settle down at the bottom for 6 hours (Fig. 1). After that, the water in the upper portion of the beaker was decanted; using filter media, it was filtered; and then the sunlight was used to dry it under controlled conditions to avoid any contamination.

### 2.3 Chemical treatment

The raw material UMP was impregnated with ZnO using solid fusion assisted by microwaves. For this, a weighed amount of raw biosorbent was sprayed onto a Petri dish, and 10 g of zinc oxide was evenly spread over the biosorbent. Distilled water was sprinkled on the mixture to facilitate solid fusion, and the mixture was then microwaved. This was then well mixed with a sterile spatula, further heated, and allowed to rest at room temperature. Subsequently, it was ground and stored in an airtight flask with the label TMP.

### 2.4 Congo red solution preparation

To check the efficiency of the biosorbent, we initially mixed 1.0 g of CR dye with a smaller volume of water, which was subsequently diluted with 1000 mL of D.W. to reach a concentration of 1000 ppm. Standards with concentrations ranging from 5 to 50 ppm were prepared using this stock solution. The absorbance of these standard solutions was measured using a spectrophotometer (721-spectrophotometer, M-ETCAL) at a wavelength of 497 nm. It was used to build a calibration curve, which, in turn, was used to determine the concentration of dye before and after the addition of the adsorbent.

#### 2.4.1 Cost analysis

The economic feasibility of the developed biosorbent was evaluated based on the cost of the raw materials, preparation, and adsorption performance. The primary precursor used in this study, *Musa paradisiaca* (banana peel), is an abundant agricultural waste material and is considered to have a negligible cost. The major cost contribution arises from the chemical reagents used for modification, such as ZnO and other processing chemicals.

In general, the cost of adsorption processes can be assessed by considering factors such as raw material cost, chemical consumption, energy requirements, and adsorption capacity. Compared with commercial adsorbents, like activated carbon, which are relatively expensive, agro-waste-derived biosorbents offer a significant economic advantage due to their low-cost and wide availability. Previous studies have reported that the use of agricultural waste materials can reduce treatment costs substantially while maintaining effective adsorption performance.

Furthermore, the adsorption capacity (153.846 mg g^−1^ for TMP) obtained in this study indicates that a relatively small amount of adsorbent is required for efficient dye removal, which further reduces the overall treatment cost. Compared with recently reported studies, the present system demonstrates a favorable balance between adsorption efficiency and material cost, making it a promising candidate for large-scale wastewater treatment applications.

In addition, the regeneration ability of the adsorbent enhances its economic viability by enabling repeated use, thereby reducing overall operational costs. Therefore, the developed ZnO-modified banana peel biosorbent can be considered a cost-effective and sustainable alternative for dye removal from wastewater. Acid is required for the regeneration step. This adsorbent is regenerated three times in laboratory conditions. As the cycle number increases, the adsorbent loses efficiency, and regeneration costs are gradually reduced. The cumulative regeneration cost for the run of three cycles is nearly 0.205 $/kg.

### 2.5 Characterization of biomaterial

#### 2.5.1 Structural evaluation

Several functional groups were identified using FT-IR analysis. Before and after adsorption, an FT-IR study of the adsorbents UMP and TMP was carried out. To ascertain whether the surface was homogeneous or heterogeneous and to look into the surfaces, SEM analysis was performed. Given the possible risks that heavy metals pose to aquatic life and human health, E. D. X. examination for sorbent was carried out to find heavy metals. Iodine number, moisture content, and ash and volatile content were used to gauge the effectiveness of the banana peels.^18^ The next calculation was used to calculate the adsorbent's dry density:1
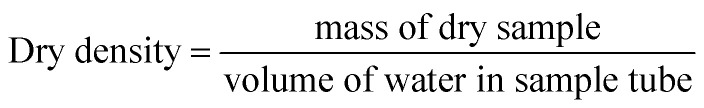


#### 2.5.2 Batch adsorption

The main objective of this investigation was to optimize a set of factors, like pH, time of contact, speed of agitation, temperature, and dose of adsorbent. The stock solution of the dye was diluted to the required concentration of 25 ppm. A series of batch adsorption tests was conducted to investigate various parameters, including pH between one and ten, temperature between twenty and eighty, speed of orbital shaking (between 60 and 140 rpm), dose of biosorbent (0.20 to 1.8 grams), time of contact between ten and one hundred minutes, and the concentration of dye at the initial stage (between 5 and 50 ppm). An Erlenmeyer flask with 0.8 g of adsorbent and 25 mL of dye solution was mechanically shaken in every experiment. The percentage of dye removal was determined using eqn (2):^19^2
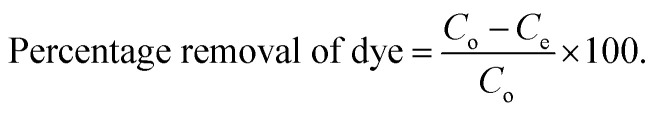
3
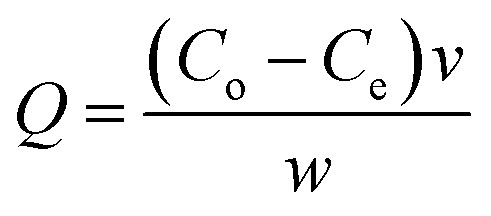


#### 2.5.3 Adsorption isotherms

The analysis of the adsorption isotherm is fundamental in establishing the interaction between the adsorbate and adsorbent in their respective phases. In order to determine a suitable method for treating industrial wastewater, experimental data of adsorption were processed with the help of different isotherm models. Adsorbed molecules are believed to interact energetically during multilayer adsorption on different adsorbent sites according to the Freundlich isotherm model. Eq-4 represents its linearized version:4
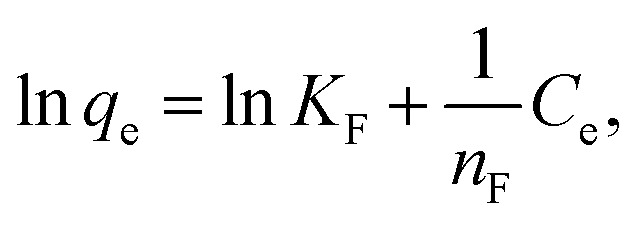
where the heterogeneity factor is denoted by (1/*n*_F_) and sorption capacity (Table 2) is linked to the Freundlich isotherm constant *K*_F_ (mg g^−1^). Here, “*K*_F_” may be calculated using the graph's intercept between ln *q*_e_ and ln *C*_e_, and the “*n*_F_” can be obtained using the slope (Table 6).

**Table 2:** Isothermal parameter description

Value	Langmuir isotherm model (*R*_L_)	Freundlich isotherm model (1/*n*_F_)
0	Irreversible	Spontaneous
1	Linear	Linear
>1	Unfavorable	Irreversible

The Langmuir isotherm model, however, considers that the molecules adsorbed do not travel across neighboring sites and that the dyes adsorb on the surface of homogenous adsorbent sites with the same energy level. Eqn (5) expresses the Langmuir isotherm model in linear form:5
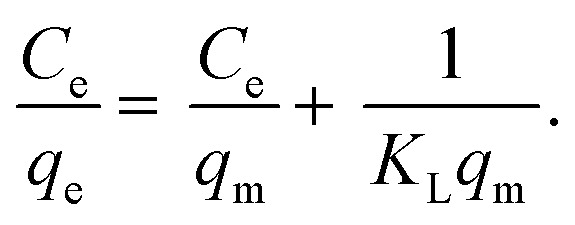


The Langmuir isotherm (L mg^−1^) is represented by *K*_L_, the adsorption capacity (mg) at equilibrium (mg g^−1^) is denoted by *q*_e_, and the maximum dye removal capacity (mg g^−1^) is denoted by *q*_m_. These values are compared with the adsorbents listed in Table 7. Plotting a graph between *C*_e_/*q*_e_*versus C*_e_, with the maximum adsorption capacity being the inverse of the slope, might be used to estimate the values of *q*_m_ and *K*_L_. *K*_L_ was then computed using the intercept.

The relationships between adsorbate molecules in the solution phase with a unique adsorbent surface are assumed by the Temkin isotherm. The idea behind this is that as the solid surface is covered, the heat of adsorption drops. Eqn (6) is used to calculate the isotherm's parameters:6
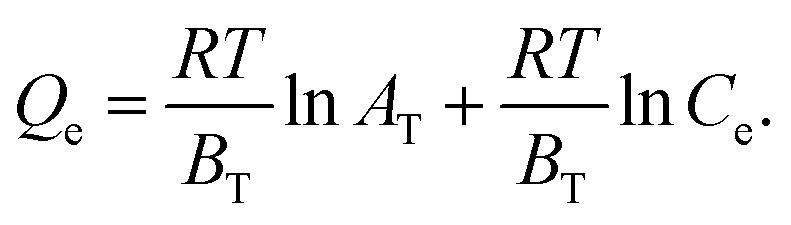


The energy of binding (*A*_T_) and heat of adsorption (*B*_T_) can be calculated from the slope and the intercept of a linearized graph.

#### 2.5.4 Adsorption kinetics

The pseudo 1st order kinetics model (eqn (7)), which states that the rate of adsorption is exactly proportional to the solute concentration, was successfully used to model the experimental adsorption data. The pseudo 2nd order kinetics model (eqn (8)), however, makes the assumption that the adsorption rate is proportional to the square of the solution concentration.:^20^7
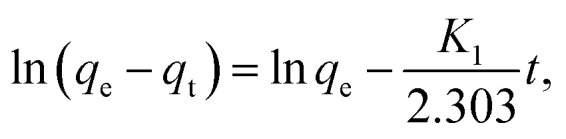
8
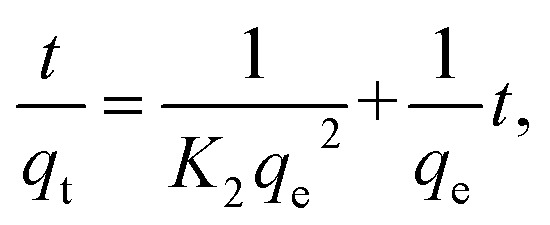
where ‘*t*’ denotes time in minutes and ‘*q*_*t*_*’* is the amount of the adsorbate adsorbed (mg g^−1^).^21^ The RMSE was calculated using eqn (9):9
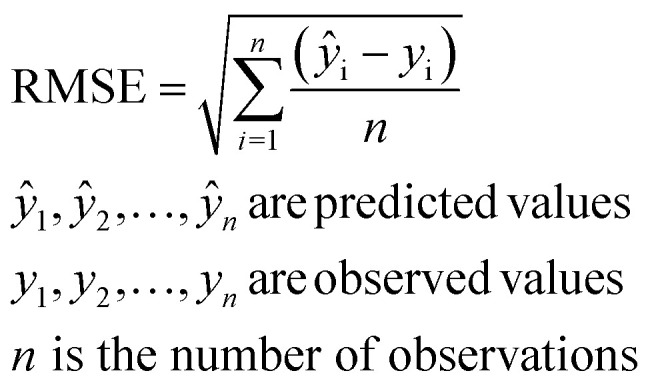


#### 2.5.5 Adsorption thermodynamics

In adsorption chemistry, process impulsiveness is mostly determined by energy considerations. By shedding light on the viability and usefulness of adsorption mechanisms, thermodynamic parameter values affect the actual application of a process. This is the van't Hoff equation (eqn (10)):10
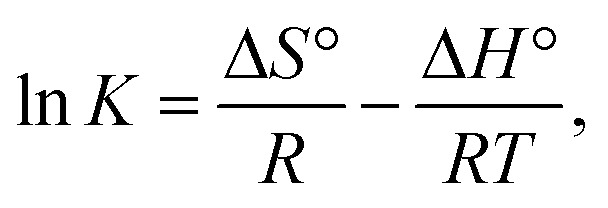
where “*T*” denotes the adsorption temperature expressed in Kelvin and “*R*” denotes the universal gas constant (8.314 J mol^−1^ K^−1^). Here, the chemical energy is equivalent to the adsorbate's Gibbs free energy (Δ*G*^°^ in kJ mol^−1^) in each of its phases. The thermodynamic connections between enthalpy (Δ*H*^°^ in kJ mol^−1^) and entropy (Δ*S*^°^ in J mol^−1^ K^−1^) can be used to calculate unrestricted energy.^22^

#### 2.5.6 Method for regeneration

0.1 M NaOH and 0.1 M HCl solutions were used to treat the sample under both basic and acidic conditions, respectively. To get rid of any leftover acid or base, the adsorbent was cleaned three times with distilled water. After 12 hours at 80 °C in an oven, the adsorbent's effectiveness is once more assessed.

## 3 Results and discussion

### 3.1 Properties of raw materials

See Table 3.

**Table 3:** Physiochemical characterization of the UMP

Property	UMP
Particle density	0.879 g cm^−3^
Bulk density	0.375 g cm^−3^
Porosity	56.10%
Moisture	8.8%
Ash	4.9%
VOCs	86.71%
I_2_ number	358 mg g^−1^

#### 3.1.1 SEM analysis

**Fig. 2:**
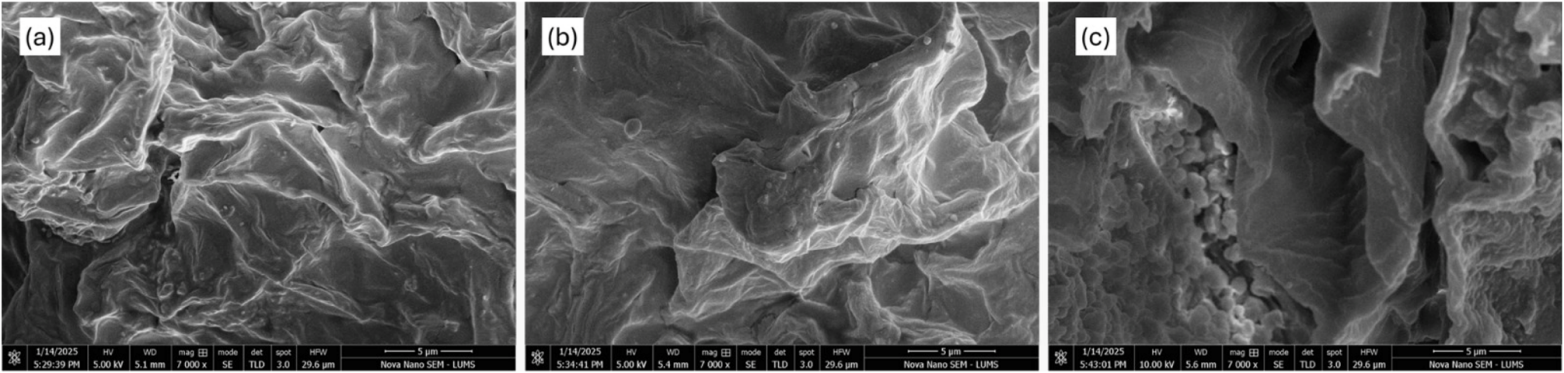
SEM analysis: (a) UMP without reaction with CR, (b) UMP after reaction with CR, and (c) TMP after reaction with CR.

Surface characterization of the biosorbent was examined using SEM–EDX, and the results are reported in the form of figures. The raw adsorbent surface is quasi-flat and exposes fewer binding sites, as shown in Fig. 2(a and b). This is further demonstrated in Fig. 2-A(c and d) following microwave-assisted ZnO treatment. Following adsorption, CR was applied to these locations, indicating that the physisorption mode of biosorption would also improve subsequent chemical treatment in addition to the biomass adsorption capacity for dye.

#### 3.1.2 EDX analysis

Banana peels have demonstrated high effectiveness for treating wastewater, both before and after ZnO modification.

**Fig. 3:**
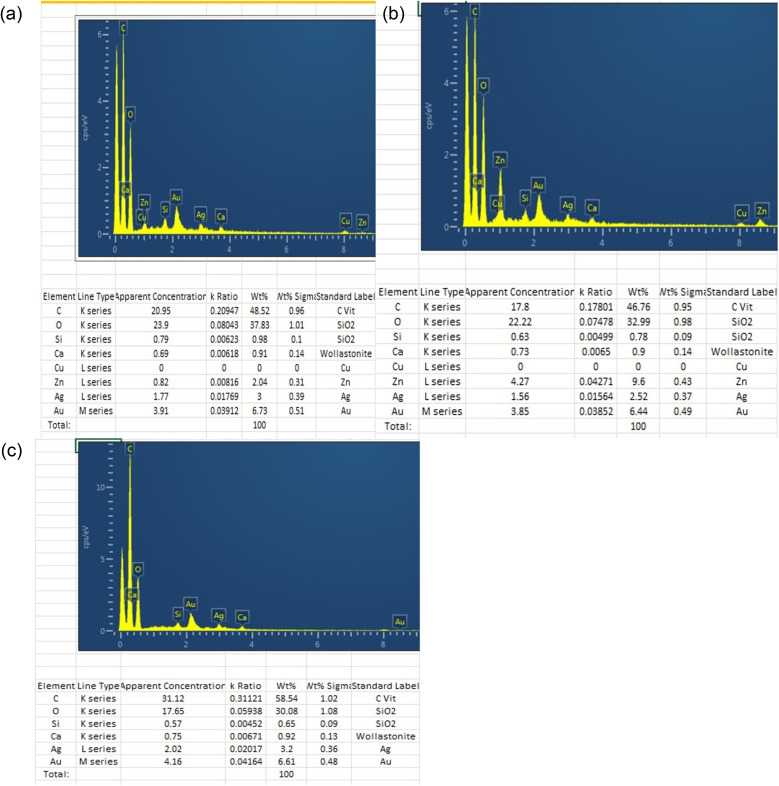
EDX analysis: (a) UMP after adsorption of CR, (b) TMP before adsorption of CR, and (c) TMP after adsorption of CR.

This shows that *Musa paradisiaca* peels are safe to use for water treatment because they do not contain any hazardous materials or metal ions (Fig. 3), even after the adsorption of CR dye, so it is feasible for larger-scale phyto filtration of dyes.

#### 3.1.3 FT-IR analysis

**Fig. 4:**
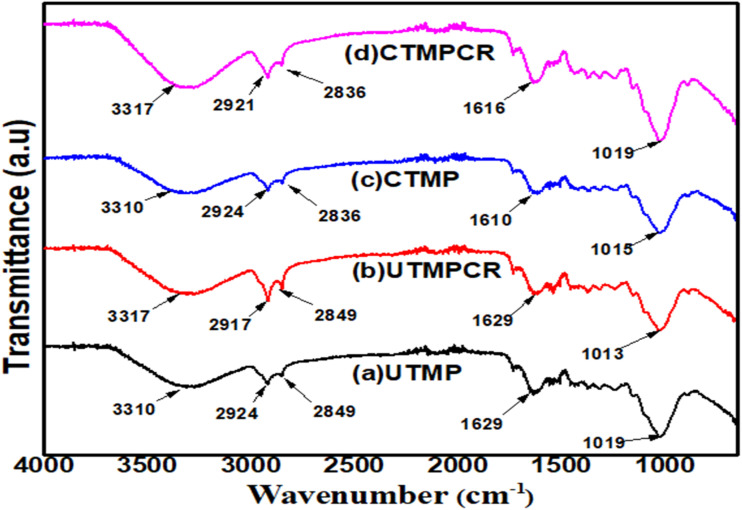
FT-IR spectra of biomaterials before and after CR dye removal.

Different functional groups are determined in the FT-IR spectra (Fig. 4) of the biomaterial samples, as illustrated in Table 4. The CR dye adsorption on TMP causes the peak values to shift slightly towards the lower wave number, which depicts that the chemisorption phenomenon is the most common in these samples. To analyze the adsorption process, we obtained the results of UMP and TMP in the raw and after the reaction with the CR dye. The names of the different functional groups and bonds were assigned according to the wavenumbers reported in the literature.

**Table 4:** Comparative FT-IR analysis of raw and CR-treated samples

Functional group	Values in IR spectrum (cm^−1^)	Raw form (cm^−1^)	After reaction (cm^−1^)
**UMP**			
C–H stretching	2800–3300	2924	2917 [ref. 23]
C–C stretching	1200–1600		
C–O stretching, O–H, C=O carboxylic	1000–1300, 3200–3600, 1600–1800	1019, 3310, 1629	1013, 3317, 1629

**TMP**			
O–H	3200–3600	3310	3317 [ref. 24]
C–H stretching	2800–3300	2924	2921
C=O carboxylic	1600–1800	1610	1616
C–H bending	500–1500		
C–C stretching	1200–1600		
C–O stretching	1000–1300	1015	1019 [ref. 25]

The FTIR spectra of untreated banana peel (UMP) and banana peel modified with ZnO (TMP) prior to and after adsorbing the Congo red (CR) dye proved the presence of various functional groups that bind to the dye. The maximum at 2924 cm ^−1^ in UMP is attributed to C–H stretching vibrations of aliphatic groups, which had slightly changed to 2917 cm^−1^ upon CR adsorption, indicating an interaction between the dye molecules and the adsorbent surface.^26^ The wide band at 3310 cm^−1^, which is the OH stretching vibration of hydroxyl groups in lignocellulosic components, changed to 3317 cm^−1^ after adsorption, indicating that hydroxyl groups were involved in hydrogen bonds with CR molecules. The maximum at 1629 cm^−1^, which is attributed to the C=O stretching of carboxylic groups, was virtually the same after adsorption, suggesting partial involvement in the adsorption process. Similarly, the C–O stretching vibration at 1019 cm^−1^ was slightly shifted by 1013 cm^−1^, and this once again supported the reaction of oxygen-containing functional groups with the dye molecules.

In the case of TMP, the O–H stretching band at 3310 cm^−1^ was shifted to 3317 cm^−1^ following CR adsorption, while the C–H stretching band at 2924 cm^−1^ was also changed to 2921 cm^−1^ following CR adsorption, indicating a surface interaction between the dye and the ZnO-modified biosorbent. The C=O carboxylic peak was also changed to 1616 cm^−1^, which shows that there is a potential electrostatic interaction between the CR dye anion and the adsorbent surface. Additionally, the C–O stretch moved to 1019 cm^−1^ instead of 1015 cm^−1^, indicating the presence of oxygen functional groups in the adsorption process. These changes in the spectra ensure that hydroxyl, carboxyl and oxygen functional groups are influential in the biosorption of CR on the UMP and TMP biosorbents.

#### 3.1.4 Zero point charge (ZPC) determination

**Fig. 5:**
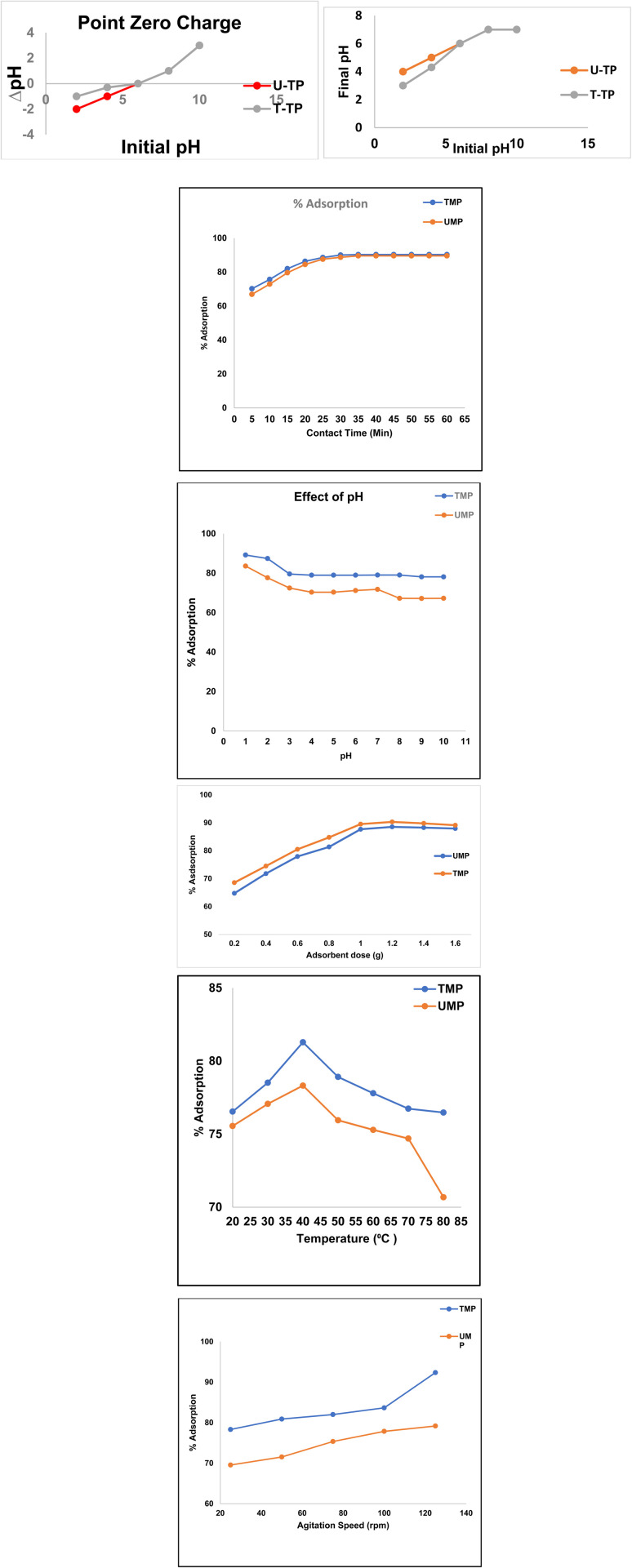
(a) Point of zero charge of the sorbent. (b) Congo red elimination in relation to contact time. (c) Effect of pH on the removal of CR dye. (d) Effect of biosorbent dosage on the adsorption of CR dye. (e) Effect of temperature on the removal of CR dye. (f) Effect of agitation speed on the removal of CR dye.

To find the banana peel's surface point of zero charge, 2 groups of 20 conical flasks, labelled 1–10 for the untreated banana peel and the same labelled for the treated banana peel, were used; ten solutions of NaCl of 25 ppm with pH ranging from one to ten were prepared by adjusting with 0.1 M hydrochloric acid and 0.1 M sodium hydroxide, and the pH was adjusted accurately using a pH meter. One group of 10 conical flasks with pH values ranging from one to ten was prepared to determine the ZPC. Each flask was then filled with a salt solution with a 25 ppm concentration of up to 200 mL and 0.5 g of untreated banana peel (UMP), and the same was repeated using the treated banana peel (TMP). The final pH of each sample was established after the samples had been allowed to remain at 25 °C for 24 hours. The visual determination of the ZPC was then done by the difference in the pH values before and after. The zero point of banana peel (UMP) was determined as 6, and zinc oxide-modified banana peels (TMP) were determined as 5.8 (Fig. 5a). Because of the interaction between the adsorbent and adsorbate, the ZPC point of the zinc oxide-modified banana peels decreased to the same level as that of the unmodified banana peel. The adsorbent surface is more positive when the pH is lower than the PZC and is more favorable for adsorbing anionic dyes, such as CR. This influences the acid–base balance of the interface of the material under aqueous conditions. Consequently, the basic dyes are easily circumvented by adsorbing anions on positively charged adsorbent surfaces. The zero charge of TMP falls within the range of the values reported in the literature,^27^ such as 5.83 and 5.0.^28^ It is easier to understand from the graph how the charge distribution changes throughout the adsorbent surfaces when ΔpH against initial pH is shown graphically. Both unmodified banana peels (UMP) and zinc oxide-modified banana peels (TMP) showed comparable values with the literature. The slight variation in the optimum pH values for UMP (pH 6) and TMP (pH 5.8) can be attributed to surface modification caused by ZnO impregnation. The presence of ZnO nanoparticles alters the surface functional groups and charge characteristics of the adsorbent, which affects the electrostatic interaction between the adsorbent surface and the anionic Congo red dye molecules. Consequently, the ZnO-modified BP exhibits slightly different optimal pH conditions for maximum adsorption efficiency compared with the untreated banana peel adsorbent.

### 3.1.5 Contact time

According to this graph, a banana peel treated with ZnO becomes an excellent sorbent for the detoxification of CR dye, showing greater sorption. Interactions between dye and UMP and TMP demonstrate the connection between binding site availability and adsorption time. A sharp rise slope was observed in both, suggesting that the dye was removed from the solution quickly. A steeper initial slope and quicker initial adsorption were observed in the banana peel treated with ZnO because of the enhanced surface area caused by the ZnO nanoparticle treatment. Additional active sites provided by ZnO improved the electrostatic attraction between ZnO and CR molecules.

The fact that the blue line continuously stays above the red line indicates that ZnO treatment greatly enhances adsorption kinetics and capacity. However, relying on natural functional groups (cellulose, lignin, and pectin), untreated banana peel exhibits strong initial adsorption but at a slower pace. Peel that has not been treated achieves lower equilibrium adsorption (about 60–75%). Both compounds showed good removal percentages at a relatively low concentration of 25 ppm although ZnO treatment increases process efficiency. The adsorbent-to-solution ratio promotes effective dye removal with just 25 mL of solution. ZnO nanoparticles improve porosity and surface area. The equilibrium adsorption percentage of ZnO-treated peel is greater (probably 85–95%). Through hydrogen bonds, electrostatic interactions, and possible cooperation with Congo red molecules, ZnO offers more binding sites. Diffusion resistance is decreased by ZnO treatment, making it easier for dye molecules to reach binding sites. According to this graph (Fig. 5b), banana peel treated with ZnO becomes a very excellent sorbent for the detoxification of CR dye, attaining greater sorption percentages in shorter periods.^29^

### 3.1.6 Effect of changes in pH

We use 0.1 M sodium hydroxide and 0.1 M hydrochloric acid solutions to stabilize the pH of the dye adsorption on the lignocellulosic surface of banana peels during the experiment. The zero-point charge was found to be 6 for untreated banana peels (UMP) and 5.8 for treated banana peels (TMP). This is essential for assessing their capacity to adsorb Congo red dye.^30^ Using a pH range of 1–10, the graph showed how pH affects dye adsorption on UMP and TMP (Fig. 5c). It is evaluated from the graph that at a lower pH, dye adsorption is greater compared to high pH, where adsorption shows a continuous decline with increasing pH.

Biosorbents exhibit greater positively charged surfaces at pH values below the zero-charge point, which is advantageous for the adsorption of anionic dyes, like Congo red, and at higher pH than PZC, the biosorbents show a negative charge surface and hence produce repulsion for anionic dyes, showing decreasing character for dye adsorption. At all pH values, the TMP showed better adsorption than the UMP because Zn^2+^ ions also showed adsorption for the anionic dye. The maximum adsorption was recorded at pH 01, where UMP showed nearly 83% adsorption and TMP showed 90% adsorption.

### 3.1.7 Biosorbent dose effect

Under diverse experimental conditions, the dosages of UMP and TMP sorbents ranged from 0.2 g to 1.6 g, with every increase of 0.2 g. Both UMP and TMP adsorbents have a peak at about 3600 cm^−1^, which indicates the presence of hydroxyl (OH^−1^) functional groups, according to FT-IR research. In particular for Congo red (CR), this suggests increased interaction with acidic dyes.

In this work, two groups of 10 Erlenmeyer flasks were used to ensure experimental investigations. Each flask held a 25 ppm solution in a volume of 25 mL. Throughout the adsorption procedure, the experimental parameters were maintained at room temperature. In accordance with the data displayed in the plot, the highest detoxification efficiency of CR adsorption from the aqueous solution for UMP at a dose of 1.20 g was 84.61%, while for TMP at a dose of 1.2 g, it was 91% (Fig. 5d). Furthermore, the graphs demonstrate that because there were more binding sites on the TMP, the adsorption of CR dye increased at lower concentrations. The tendency of the adsorbent molecule to aggregate at greater concentrations, however, occluded these binding sites and caused a quick equilibrium between the adsorbents and adsorbates,^31^ which in turn reduced overall adsorption. The highest effective adsorbent mass for TMP and UMP, according to additional experimental research, was 1.2 g.

### 3.1.8 Temperature effect

The temperature range (20–80 °C) was used to examine the effect of temperature on the adsorption of the CR dye. With maximum adsorption of 77.84% by UMP and 82.40% by TMP recorded at 40 °C, the results show that dye removal utilizing unmodified banana peels (UMP) and treated banana peels (TMP) rises with temperature up to 40 °C; then, further increase in temperature results in decrease in adsorption (Fig. 5e). This study demonstrates that the adsorption process is endothermic until 40 °C and then becomes exothermic as higher temperatures enhance the removal of the target dye by desorption from banana peel.^32^

The observed drop in the effectiveness of dye removal at higher temperatures might be the result of the boundary layer becoming narrower, which makes it more likely for molecules to escape into the solution from the adsorbent surface. Furthermore, the contact time required for effective adsorption of the target material is decreased by improved particle mobility at higher temperatures. As the temperature rises, this leads to a reduction in adsorption capability.

### 3.1.9 Agitation speed effect

By promoting the interface between the positively charged surface of the sorbent and the anionic molecules of the dye, an ideal agitation rate increases the efficacy of adsorption by creating a distinct outer border. Higher agitation rates result in more binding sites being available,^33^ which also prevents a barrier from forming between the dye and the adsorbent. This causes an increase in adsorption. At rates between 25 and 125 rpm, this research examined the impact of agitation speed on CR adhesion. The findings are displayed in Fig. 5f.

Other optimum parameters for each dye solution employed in the batch analysis were a concentration of 25 ppm, 1.2 g adsorbent dosage, a temperature of 40 °C, a shaking speed of 130 rpm and a contact period of 40 minutes.

### 3.1.10 Thermodynamic studies

**Fig. 6:**
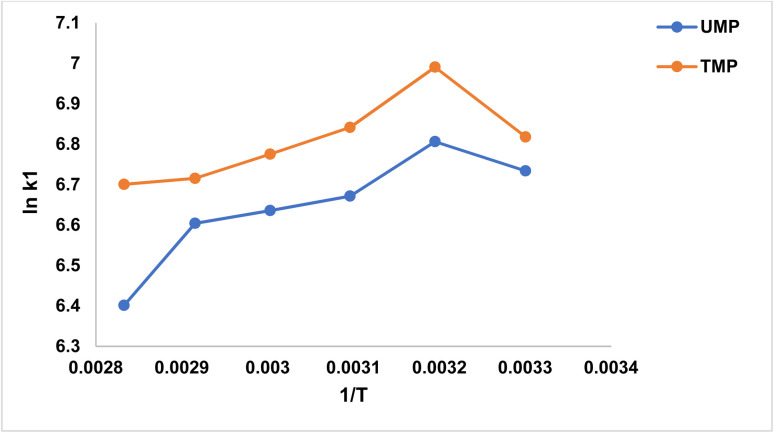
Thermodynamic graph of CR dye removal by UMP and TMP.

The experimental analysis of the parameters of CR dye adsorption was conducted, and the parameters were optimized with respect to temperature variation, where a close relationship was established between temperature and adsorption. The findings have shown that color removal increases as temperature increases up to 77.84% by UMP and 82.40% by TMP recorded at 40 °C. The results show that dye removal utilizing unmodified banana peels (UMP) and treated banana peels (TMP) increases with temperature until 40 °C; then, a subsequent increase in temperature results in a decrease in adsorption. This study demonstrates that the adsorption process is endothermic up to 40 °C and then becomes exothermic as higher temperatures enhance the elimination of the selected dye by desorption from banana peel. For banana peels, high temperatures change the adsorbent morphology by expanding, protruding, and elongating the particles, leading to enhanced dye desorption because of the deteriorating structure of the adsorbent. These structural defects enable CR dye to desorb from defective areas, leading to a decreased rate of adsorption. Notably, dye desorption on TMP occurs less effectively than on UMP from 40 to 80 degrees Celsius. The cellulosic substrate has porosity-enhanced frameworks, and it is sensitive to changes in temperature, which influences the adsorption and mechanistic energy of CR, thereby increasing the rate of dispersion. Some significant thermodynamic features, provided in Table 5 and depicted in Fig. 6, determine the effectiveness and adsorption rate of dyes on biosorbents, such as UMP and TMP. These features vary with thermodynamic parameters.^32^ Lower RMSE values signify a highly controlled and accurate chemical fit in the case of TMP.

**Table 5:** Thermodynamic evaluation of CR adsorption by UMP and TMP

Biosorbent	*T* (K)	Slope	Intercept	*R* ^2^	RMSE	Δ*G*° (kJ mol^−1^)	Δ*H*° (kJ mol^−1^)	Δ*S*° (J mol^−1^ K^−1^)
UMP	303	3120.39	−2.4087	0.92368	0.086	−16.964	−25.943	−20.026
	313					−17.712		
	323					−17.916		
	333					−18.372		
	343					−18.833		
	353					−18.788		
TMP	303	2481.19	−0.4569	0.97188	0.048	−17.175	−20.628	−3.799
	313					−18.192		
	323					−18.373		
	333					−18.758		
	343					−19.151		
	353					−19.665		

It is worth noting that adsorbents treated with ZnO had a faster, exothermic, and spontaneous adsorption process. In this case, the decrease in the value of Δ*S* indicates that randomness decreases as structural changes occur. Entropy values ranging from 2 to 20 kJ mol^−1^ imply the existence of a strong surface, leading to physiosorption. Additionally, the enthalpy change values of −25.9432 and −20.6285 kJ mol^−1^ for UMP and TMP, respectively, further support the concept of physiosorption. The enthalpy is negative, which implies that the reaction is exothermic. These thermodynamic values were calculated using the following formula: Δ*G*° = Δ*H*° − *T*Δ*S*°.^34^ The calculated enthalpy change values were −25.94 kJ mol^−1^ for UMP and −20.63 kJ mol^−1^ for TMP. These values fall within the range generally associated with physical adsorption, indicating that the adsorption process is predominantly governed by physisorption mechanisms, such as electrostatic interactions and hydrogen bonding. The negative Δ*H*° values further confirm the exothermic nature of the adsorption process.

The chemical study for the adsorption of each CR on UMP and TMP was analyzed, and the findings are illustrated in the Table 5. The substantial negative values signify a strong adsorption rate. The graph in Fig. 6 represents linear correlation between “ln *K*_D_” and “1/*T*”.^35^

### 3.1.11 Adsorption isotherm studies

**Fig. 7:**
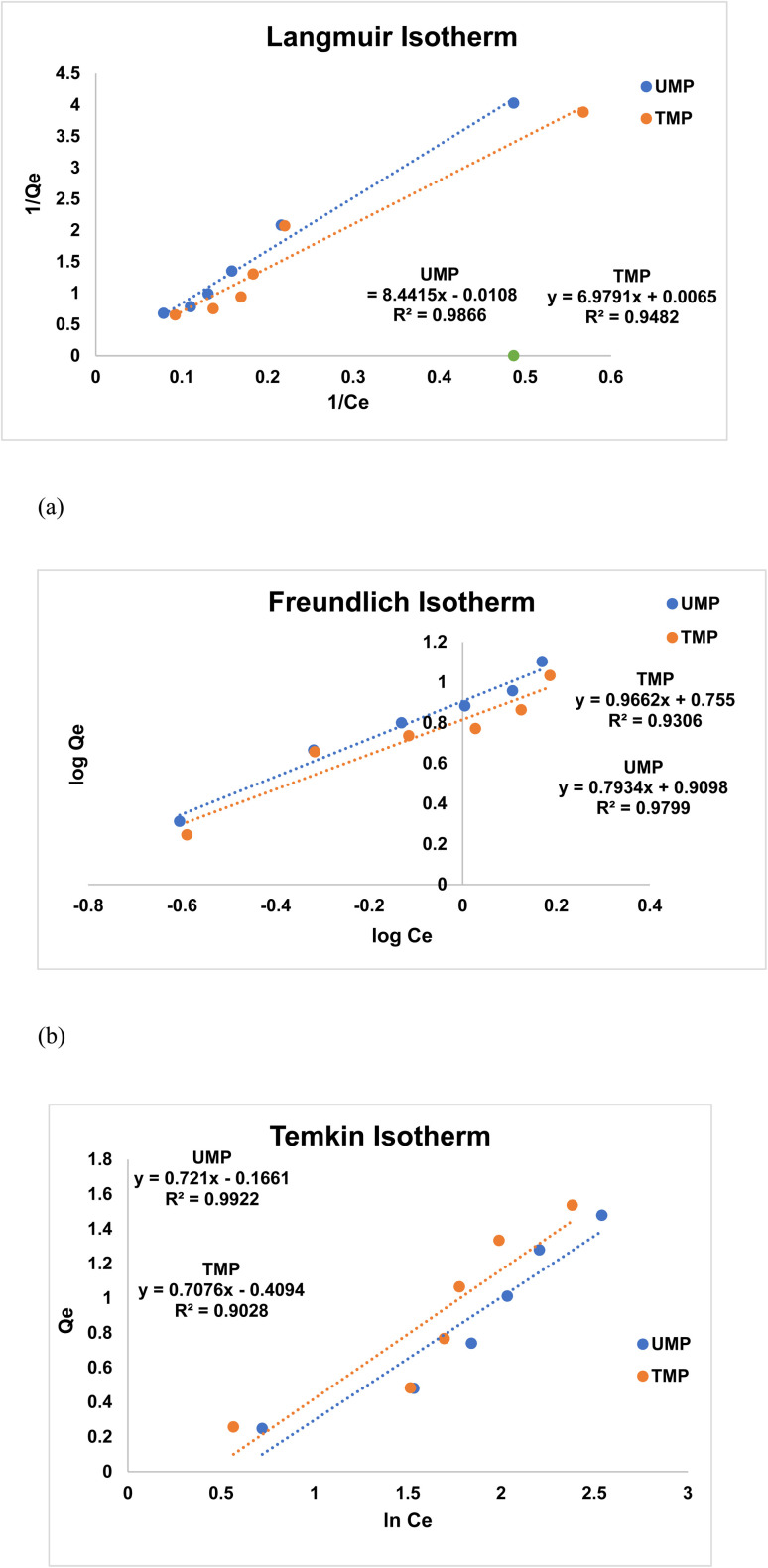
Comparison between isothermal models for unmodified banana peel (UMP) and zinc oxide-modified biosorbent (TMP): (a) Langmuir, (b) Freundlich and (c) Temkin models.

To demonstrate the associated behavior and determine the maximum *q*_max_ for unmodified and modified banana peels for Congo red dye, Langmuir, Freundlich, and Temkin isotherms are used, as shown in Fig. 7, with the corresponding factors shown in Table 6. The adsorption mechanism, surface characteristics, and adsorbent attraction to Congo red (CR) from hydrous solutions were all noted by applying different isothermal models. For untreated banana peels (UMP), the Temkin model has an *R*^2^ value of 0.9922, which is the best among all. The Langmuir model with an *R*^2^ value of 0.9482 provides the best fit, supporting the sorption of CR on treated banana peels (TMP) with ZnO outperforming the other two models.^36^ The model fitting improved greatly after the chemical treatment of the peels. The Langmuir model showed a significant decrease in RMSE from 0.0421 to 0.0115 mg g^−1^. This means that the activation process managed to homogenize the surface of the peels by stripping them of impurities. This resulted in more “ideal” adsorption through the formation of uniform monolayers, which is consistent with the assumptions of the Langmuir theory. The Temkin model had the highest RMSE (0.3954 for UMP and 0.2817 for treated ones), especially when considering the unmodified samples. Therefore, the biosorption on ZnOBP did not follow the multi-layer heterogeneous nature of adsorption. The relatively higher RMSE of the Freundlich and Temkin models can be attributed to the presence of functional groups on the surface of the peels.

**Table 6:** Comparison of isothermal conditions for the removal of CR from TMP and UMP

Parameter	UMP	TMP
**Langmuir isotherm parameters**		
*R* ^2^	0.9866	0.9482
RMSE	0.0421	0.0115
*Q*_max_ (mg g^−1^)	92.593	153.846
*R*_L_	0.978	0.976

**Freundlich isotherm parameters**		
*R* ^2^	0.9799	0.9306
RMSE	0.1853	0.1421
*n* (1/*m*)	1.2603	1.0349

**Temkin isotherm adsorption model**		
*R* ^2^	0.9922	0.9028
RMSE	0.3954	0.2817
*K*_T_ (L g^−1^)	1.2590	2.2406
*B*_T_ (kJ mol^−1^)	0.721	0.8338

Even though classical isotherm models, like Langmuir and Temkin, help understand the behavior of adsorption, they simplify the surface properties and fail to explain the surface heterogeneity. Recent research has demonstrated that statistical physics-based models have the potential to give a much more detailed description of the adsorption processes by including parameters like the energy distribution of adsorption and site density and can thus connect macroscopic observations to microscopic mechanisms. These methods permit further insight into surface heterogeneity and adsorption energetics. However, this high-level modeling needs further experimental and computational analysis, which is beyond the scope of the current research. These strategies can be used in future studies to further understand the mechanism of adsorption of ZnO-modified biosorbents.

Compared with previously reported biosorbents, such as raw banana peel, Fe_3_O_4_-loaded papaya seed powder, and microbial biomass, the ZnO-modified *Musa paradisiaca* peel developed in this study demonstrates improved adsorption performance compared with the untreated material. The incorporation of ZnO provides additional active sites and enhances surface interactions with Congo red dye while maintaining the advantages of a low-cost and sustainable biosorbent derived from agricultural waste.

### 3.1.12 Kinetic studies

The adsorption kinetics were modeled using two widely used techniques (pseudo first order and pseudo second order models), with the outcomes recorded in Table 8. The evaluated parameters are shown in the table together with the corresponding rate constants (*k*_1_ and *k*_2_), equilibrium adsorption capacity (*q*_e_), and linear regression coefficients *R*^2^. The suitability of each kinetic model is determined by the correlation coefficients *R*^2^ and the consistency between the estimated and experimental *q*_e_ values. Based on these considerations, we conclude that the mechanism of pseudo 2nd order is prevalent, supported by previously published studies.^60^

**Table 7:** Comparison of CR adsorption capacities with reported biomaterials

Adsorbents	*Q*_max_ Congo red (mg g^−1^)	Reference
Euroamerican poplar	3.3	37
Algae biorefinery waste	6.20–7.28	38
Industrial hemp (*Cannabis sativa* L.)	4.47	39
*Cereus* sp.	27.02	40
Biorefinery waste of *Sargassum latifolium*	20.97	41
Polyacrylonitrile (PAN)-supported biosorbent *Moringa oleifera*	52	42
*Agaricus bisporus*	76.412	43
Sunflower seed waste	15.5	44
Jute fabric	12.863	45
Magnetic Fe_3_O_4_-loaded papaya seed powder (Fe_3_O_4_-PSP)	216.9	46
Banana peel powder	164.6	47
Euroamerican poplar	3.3	37
*Aspergillus fumigatus* and *Pseudomonas putida*	316.46	48
*Pleurotus mutilus*	36.68	49
Crab shell	124.9	50
Jujube shell	80.49	51
Peanut husk	13.5	52
*Antigonon leptopus* leaf powder	18.18	53
*Trichoderma*	81.82	54
*Vernonia amygdalina* leaf powder (VALP)	57.47	55
Fennel seed and garlic peels	15.22 and 172.43	56
Powder of cuttlefish bone	69.9	57
*Carpobrotus edulis* plant	56.18	58
*Streptomyces fradiae* biomass	46.64	59
Untreated *Musa paradisiaca*	92.5925	This study
ZnO-treated *Musa paradisiaca*	153.846	This study

**Table 8:** Comparison of kinetic equilibrium models

Parameters	UMP	TMP
**Pseudo 1storder kinetics**		
*R* ^2^	0.8157	0.8629
RMSE	0.7952	0.4812
*q*_e_	1.252	1.769
*k*_1_	0.0474	0.1193

**Pseudo 2nd order kinetics**		
*R* ^2^	0.9994	0.9994
RMSE	0.2963	0.1816
*q*_e_	5.845	5.851
*k*_2_	0.0808	0.0253

**Fig. 8:**
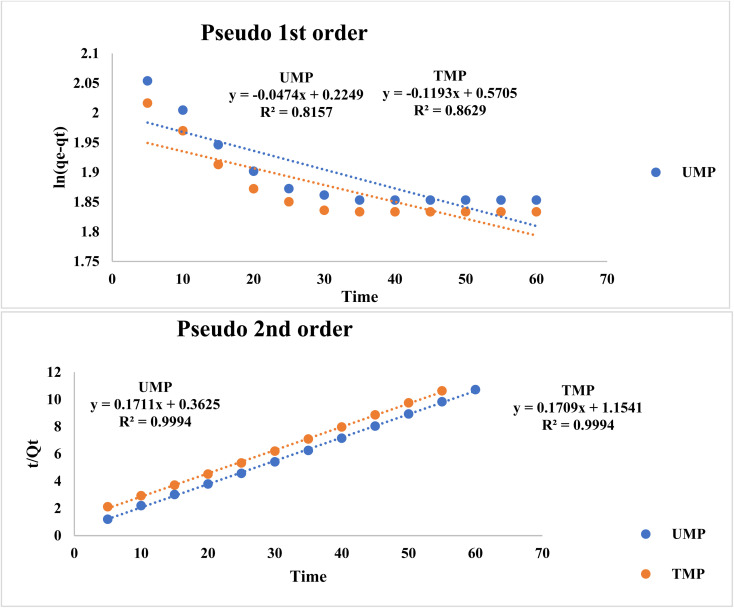
Kinetic study of the equilibrium data (a) pseudo 1st order and (b) pseudo 2nd order.

It should be noted that the model parameters presented in this study are reported without associated uncertainty indicators, such as standard errors or confidence intervals. Although the coefficient of determination (*R*^2^ in Fig. 8) provides an initial assessment of model fit, the inclusion of statistical error estimates further improves the reliability and robustness of the analysis. Lower RMSE in the case of the pseudo-second order model indicates that this model fits better than the others in TMP.

The adsorption of the research compounds (UMP and TMP) is pseudo 2nd order, with chemisorption as the rate-limiting step. This entails the exchange of electrons between adsorbent surfaces and molecules of the adsorbates, resulting in a strong interaction and maximum adsorption capacity.

**Fig. 9:**
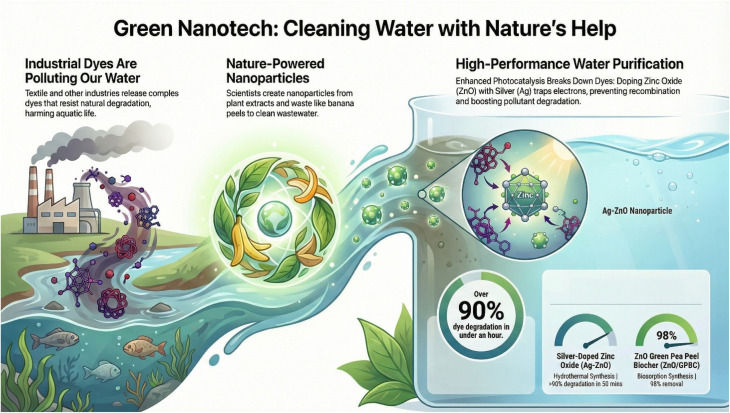
Metal oxide composites with agro-waste reported in the literature for cleaning water.

As summarized in Table 7 and Fig. 9, reported adsorbents for CR are mainly focused on the development of sustainable water treatment systems using agricultural waste and green nanotechnology. Researchers have focused on using materials such as jackfruit peels, banana plant waste, and aloe vera to produce activated carbon or bio-composites for the filtration of toxic pollutants. These bio-adsorbents are usually doped with zinc oxide or iron nanoparticles to enhance the removal of toxic dyes and pharmaceuticals from wastewater. This work assesses the efficiency of these systems using isotherm models and kinetic analysis to optimize parameters. All of them emphasize the need for eco-friendly and cost-effective alternatives to existing industrial purification technologies.

Moreover, in this study, the coefficient of determination (*R*^2^) was used as the primary statistical parameter to evaluate the fit of the adsorption models due to its ability to represent the correlation between the experimental and predicted values. Although *R*^2^ is commonly used in adsorption studies for preliminary model comparison, additional statistical errors like chi-square (*χ*^2^) and Akaike information criterion (AIC) can also be applied to provide a more rigorous evaluation of model performance. The selection of the best-fitting models in this study was therefore supported not only by high *R*^2^ values but also by the consistency of the adsorption behavior with the theoretical assumptions of the respective models.

### 3.1.13 Adsorption mechanism

The untreated and treated banana peels in terms of their surface chemistry and their attachment to Congo red (CR) were investigated by examining the SEM and FT-IR prior to and following CR adsorption. Many functional groups, such as hydroxyl group, carbonyl (C=O) and carboxyl (CO), were identified on the interfaces of UMP and TMP. The peak of banana peel at 3280 cm^−1^, corresponding to –OH groups or C–O bonds, is associated with adsorbed water molecules or intermolecularly bonded phenolic and alcoholic groups.^61^

**Fig. 10:**
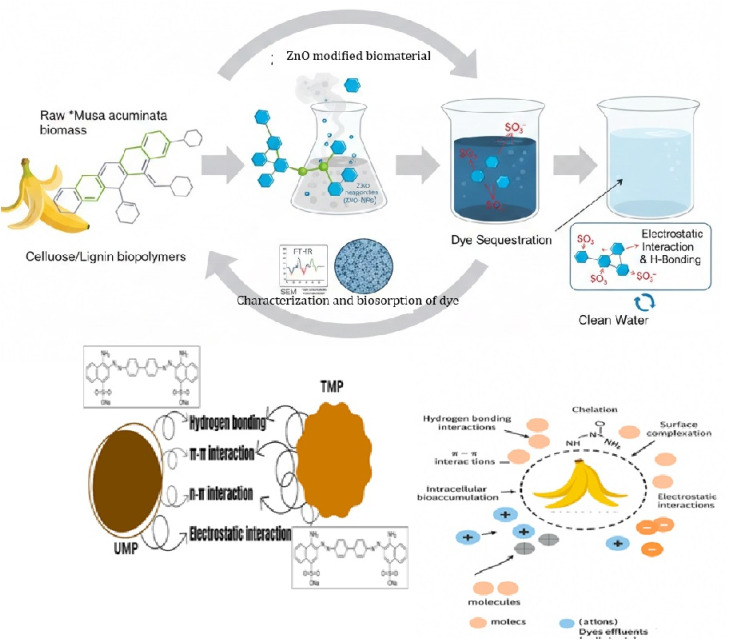
Biosorption phenomenon between CR and the biomaterial.

The oxygen atoms of these functional groups (Fig. 10) could establish H bonding with the NH_2_ groups of CR. The peaks of adsorbed CR were stronger than those of the used adsorbent because their mass is significantly less; the band around 1600 cm^−1^ is associated with extending vibrations of C=O after adsorption as well as C=N bonds of CR. Besides, the ‘O’ atom within the C=O carboxylate group within the CR may be involved in H-bonding with NH_2_ groups of CR. Electrostatic forces among the positively charged surface of CR and the negatively charged surface of banana peels are attributed to be the main adsorption mechanism in banana peels.^62^

### 3.1.14 Regeneration process

**Fig. 11:**
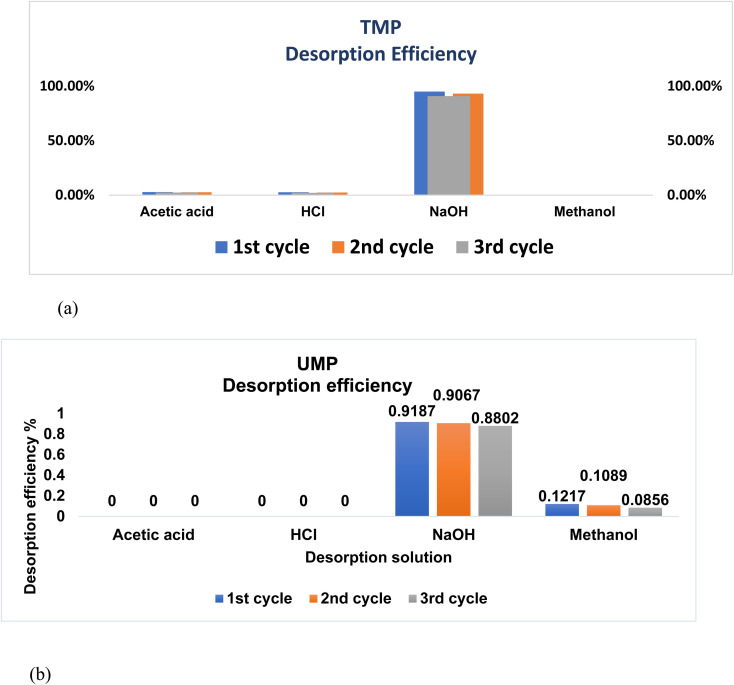
Impact of different concentrations of the desorption solution on CR: (a) TMP and (b) UMP.

To assess the desorption efficacy, several eluents were utilized to remove CR dye from the surface of the adsorbent and to disrupt the adsorbate–adsorbent interactions to use later. As depicted in Fig. 11, 0.1 M NaOH is the most effective desorbing agent, which has been selected to obtain CR desorption with a maximum efficiency of 95.87% for treated peels and 97.87% for unmodified peels (Table 9). The more the cycle of the biomass, the less the biosorptive activity it exhibited.

**Table 9:** Recycling tests after the desorption of CR

Eluent	1st cycle	2nd cycle	3rd cycle
**UMP**			
Acetic acid	0.00%	0.00%	0.00%
HCl	0.00%	0.00%	0.00%
NaOH	91.87%	90.67%	88.02%
Methanol	12.17%	10.89%	8.56%

**TMP**			
Acetic acid	2.78%	2.56%	2.34%
HCl	2.56%	2.34%	2.05%
NaOH	94.87%	93.04%	90.76%
Methanol	0.00%	0.00%	0.00%

After repeated adsorption–desorption cycles, the exhausted adsorbent should be safely managed to avoid secondary environmental contamination. As reported in previous studies,^63^ dye-loaded biosorbents can be disposed of through controlled incineration or incorporation into solid waste management systems, where the adsorbed dye molecules remain immobilized and do not leach back into the environment. Such disposal practices have been widely recommended for spent biosorbents used in wastewater treatment.

## 4 Conclusion

Bioremediation was performed using an eco composite material composed of ZnO-treated peels on the Congo red dye. ZnO is very dominant in adsorption and is easily obtainable without negative effects. As the adsorption analysis showed, the TMP had a large adsorption potential with regards to the anionic dye CR. The sorption kinetics best fitted to the pseudo 2nd order; for unmodified banana peels (UMP), the Temkin model had the best *R*^2^ value, with 0.9922 being the best of all. The Langmuir model, with the *R*^2^ value of 0.9482, is considered the best fit, supporting the sorption of Congo red by ZnO-treated TMP compared to the other two models. The thermodynamic studies confirmed that the sorption of Congo red is exothermic and spontaneous. This is further added value through the regeneration of adsorbent, and this will not only eliminate the problem of large waste generated because of banana peels but will also eliminate the threat posed by the dangerous dye present in wastewater. Thus, one possible raw material to make is banana peel, which can eliminate anionic dye impurities, such as CR dye.

## Conflicts of interest

The authors declare that they have no conflicts of interest.

## Abbreviations

UMP: Untreated *Musa paradisiaca*
TMP: Treated *Musa paradisiaca*
CR: Congo red
DW: Distilled water

## Data Availability

Data are available from the authors upon request.
